# Fixation of the fully hydroxyapatite-coated Corail stem implanted due to femoral neck fracture: 38 patients followed for 2 years with RSA and DEXA

**DOI:** 10.3109/17453674.2011.641107

**Published:** 2012-04-24

**Authors:** Thord von Schewelov, Henrik Ahlborg, Lennart Sanzén, Jack Besjakov, Åke Carlsson

**Affiliations:** ^1^Department of Orthopaedics; ^2^Diagnostic Radiology, Lund University, Department of Orthopaedics, Skåne University Hospital, Malmö, Sweden

## Abstract

**Background:**

Today, dislocated femoral neck fractures are commonly treated with a cemented hip arthroplasty. However, cementing of the femoral component may lead to adverse effects and even death. Uncemented stems may lower these risks and hydroxyapatite (HA) coating may enhance integration, but prosthetic stability and clinical outcome in patients with osteoporotic bone have not been fully explored. We therefore studied fixation and clinical outcome in patients who had had a femoral neck fracture and who had received a fully HA-coated stem prosthesis.

**Patients and methods:**

50 patients with a dislocated femoral neck fracture were operated with the fully HA-coated Corail total or hemiarthroplasty. 38 patients, mean age 81 (70–96) years, were followed for 24 months with conventional radiographs, RSA, DEXA, and for clinical outcome.

**Results:**

31 of the 38 implants moved statistically significantly up to 3 months, mainly distally, mean 2.7 mm (max. 20 mm (SD 4.3)), and rotated into retroversion mean 3.3º (–1.8 to 17) (SD 4.3) and then appeared to stabilize. Distal stem migration was more pronounced if the stem was deemed to be too small. There was no correlation between BMD and stem migration. The migration did not result in any clinically adverse effects.

**Interpretation:**

The fully hydroxyapatite-coated Corail stem migrates during the first 3 months, but clinical outcome appears to be good, without any adverse events.

Several studies have indicated that fractures that are dislocated in patients who are more than 65–70 years old are best treated with hemi- or total hip arthroplasty. In large patient materials, an increase in mortality rate has been observed during the first year for cemented arthroplasties relative to uncemented arthroplasties (Khan et al. 2002, Rogmark et al. 2002, Parker and Gurusamy 2004). Embolization of fat, bone marrow, and cement particles is thought to contribute to this (Christie et al. 1995, Elmaraghy et al. 1998, Clark et al. 2001, Riding et al. 2004, Panesar et al. 2009). There are, however, concerns regarding the fixation of the uncemented stem to the osteoporotic bone. We therefore studied the migration pattern in relation to bone mineral density and size and position of the uncemented, fully hydroxyapatite coated Corail stem in patients with dislocated femoral neck fractures.

## Patients and methods

50 patients who were admitted to our hospital between 2006 and 2008 with a dislocated femoral neck fracture, Garden type III or IV, met the inclusion criteria and accepted to participate in the study after having received verbal and written information about it. The ethics committee of Lund University approved the study (LU 33/2006).

The patients had to be mentally clear and ambulatory, with or without support. The fracture had to be obtained within 1 week prior to admission after a low-energy fall. Patients with hip disease, such as arthritis of the affected joint, were excluded. Patients were only included once. Patients aged 69–79 received an uncemented stem and a cemented cup whereas patients older than 79 received an uncemented stem with a bi-articular hemiarthroplasty head.

**Figure 1.**
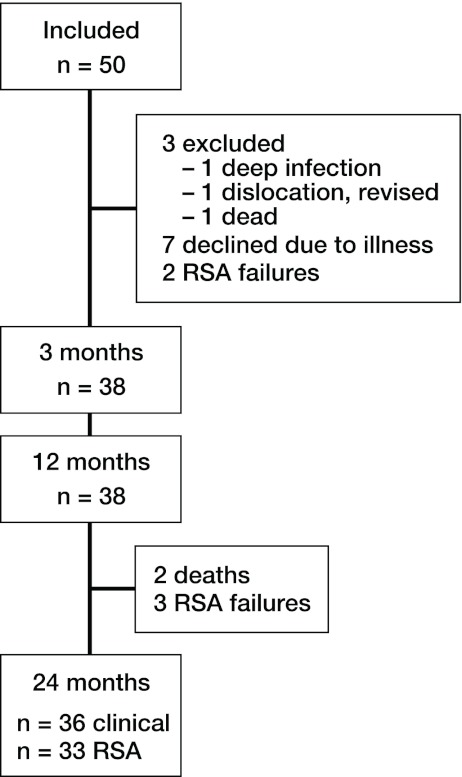
Flow chart of patients.

At 3 months, 38 patients remained (25 women) (Figure 1). Their mean age at the time of operation was 81 (70–96) years. Mean age for the 16 patients who received a total hip arthroplasty was 76 (70–79) years, and it was 85 (80–96) for the 22 patients who received a hemiarthroplasty. Prior to the fracture, 9 of the 38 patients had used a cane. 1 patient had very poor ambulatory status secondary to stroke, and therefore used a Zimmer frame. 5 patients had a disease that may have affected walking preoperatively. All patients were living at home and only 4 patients needed assistance for their daily living.

Examinations within 1 week of surgery and at 3, 12, and 24 months included clinical assessment, and radiographic and radiostereometric analysis. DEXA analysis was performed on the contralateral hip during the postoperative hospital stay, using a Lunar Prodigy (GE Healthcare, Diegem, Belgium). 3 patients with a hip prosthesis in the contralateral hip were excluded from the DEXA analysis. For the clinical evaluation, we used Harris hip score (HHS) and the Charnley score. At the 3-, 12-, and 24-month examinations a nurse, not related to the study, recorded the use of walking aids, home situation, and use of corticosteroids or drugs for osteoporosis. Pain at rest and with weight bearing in the thigh and in the hip was recorded by the patients (VAS: with 0 signifying no pain and 10 signifying the worst possible pain).

The radiographic examination included an AP view of the hip and pelvis and a lateral view of the hip. The radiographs were examined by 2 of the authors (TvS and JB) who agreed on whether or not the stem was too small relative to the proximal femoral canal. Stem position within the femoral canal was recorded—neutral or with a deviation of > 5º in varus or valgus. Stem fixation over time was evaluated using the Engh score (Engh et al. 1990).

2 of the 38 patients died between the 12- and 24-month follow-ups and were therefore monitored at the last 12-month control. Thus, 36 patients remained for clinical and radiographic examination at 24 months, but for practical reasons only 33 patients could be included in the RSA analysis.

### Prosthesis and operative technique

4 surgeons performed the operations in a clean air enclosure. Prophylaxis with systemic antibiotics was used routinely. The hips were exposed in the supine position through an anterolateral Hardinge approach without trochanteric osteotomy.

In all cases, an uncemented collarless Corail stem was used. This is a double-tapered titanium stem that is fully coated with 150 μm hydroxyapatite (HA). In patients less than 80 years old, we used a cemented Elite Plus Ogee Enduron socket with 40–47 mm outer diameter and a 28-mm modular head made of cobalt-chrome alloy. Patients older than 79 received a Monk bi-articular hemiarthroplasty head in the size range 44–54 mm. This head consists of a preassembled 26-mm head inside a polyethylene head which is covered by a shell of stainless steel articulating against the acetabular cartilage (all components were from DePuy Johnson and Johnson, Warsaw, IN). Preoperative planning was done using the mdesc digitized preoperative planning program (RSA Biomedical, Umeå, Sweden).

### Radiostereometric analysis (RSA)

**Figure 2.**
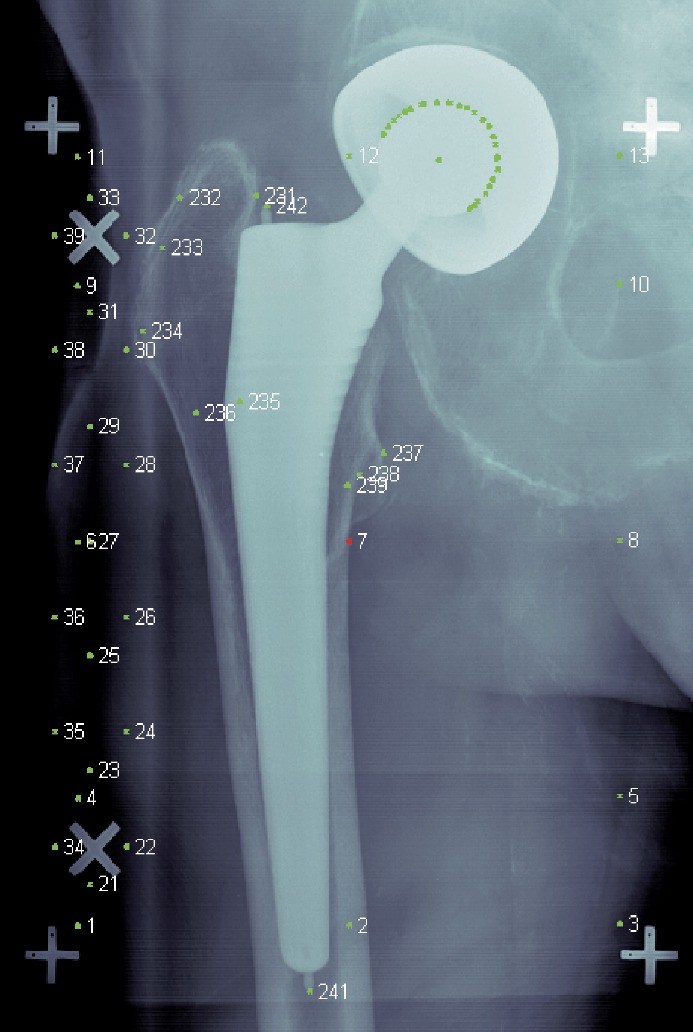
UmRSA radiograph of the Corail stem with a bi-articular Monk head. About 8 tantalum beads, 0.8 mm in diameter, were inserted in the bone (nos. 232–239). A tantalum bead of 0.8 mm in diameter was mounted on the tip of the stem (241) and at the shoulder of the stem (242). These 2 beads and the head were used to calculate stem configuration. In the case of hemiarthroplasty, the 26-mm head inside the Monk bi-articular head was visible and used. Cage markers: 1–40.

The implant and markers are shown in Figure 2. The RSA examinations were performed with the patient in the supine position using the uniplanar technique (Valstar et al. 2005). The first examination was done before the patients were mobilized to weight bearing. A second examination was done before the patient left the hospital, and thereafter 3, 12, and 24 months postoperatively. Migration and rotation of the stem were evaluated relative to the femoral bone. The radiographs were measured and analyzed using UmRSA Digital Measure (RSA Biomedical, Umeå, Sweden). Limits for statistically significant movements over time with 99% confidence intervals, were calculated using 30 double examinations. The limits were ± 0.1 mm for the longitudinal and transverse axes and ± 0.2 mm along the sagittal axis. Limits for rotation around the transverse and sagittal axes were 0.2 degrees, and 0.7 degrees around the longitudinal axis.

### Statistics

We used paired Student t-test for dependent samples and Mann-Whitney U-test. Linear regression was used when determining the association between stem migration and BMD.

## Results

### Clinical results

2 patients, who were excluded, had early deep infection and recurrent dislocation, respectively. No other complications of surgery were recorded. HHS for pain averaged 41 points out of 44 possible already at 3 months, and was unchanged after 1 and 2 years. The Charnley score for pain averaged 5.5–5.6 out of a maximum of 6 at the corresponding time points (Table).

**Table:** Pain scores. Values are mean (SD)

Months	VAS: hip		VAS: thigh		Harris hip score		Charnley score		
Postop.	Rest	WB	Rest	WB	Pain	Total	Pain	Walking	ROM
3 months (n = 38)	0.2 (0.5)	1.5 (2.0)	0.5 (1.1)	1.0 (1.5)	41 (5)	81 (12)	5.6 (0.7)	4.4 (1.5)	5.0 (1.6)
12 months (n = 38)	0.2 (0.5)	1.3 (2.6)	0.2 (0.7)	0.6 (1.7)	41 (6)	84 (13)	5.5 (1.1)	5.0 (1.0)	5.6 (1.0)
24 months (n = 36)	0.1 (0.2)	0.5 (1.2)	0.0 (0.2)	0.0 (0.2)	41 (6)	87 (21)	5.6 (0.8)	4.8 (1.6)	5.9 (0.4)

WB: weight bearing; ROM: range of movement.

**Figure 3.**
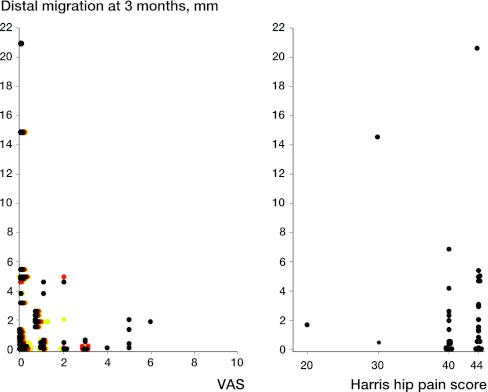
A. Pain reported on the VAS scale and distal migration at 3 months. Hip pain at rest is shown by black dots, and weight bearing by green. Thigh pain at rest is shown in red and weight bearing in yellow. B. Harris hip (HH) pain score and distal migration at 3 months. A Harris hip score of 40 means mild pain, and 44 means no pain.

At 3 months, the Charnley score for walking averaged 4.0 for hemiarthroplasties and 5.0 for total arthroplasties (p = 0.01, Mann-Whitney U-test). The corresponding values at 24 months were 4.2 and 5.8 (p = 0.001). Total HHS also differed significantly between the groups and averaged 83 and 91, respectively, at 24 months (p = 0.01). There were no statistically significant differences in Charnley pain or ROM score, nor in HH pain score, between the hemiarthroplasty group and the total arthroplasty group (p = 0.06–0.9). There was no correlation between distal migration and VAS for thigh pain at 3 months (p < 0.005, Wilcoxon matched-pair test) (Figure 3).

13 of the 28 patients who had not used a cane preoperatively used one 12 months after surgery, the majority (12) being patients with a hemiarthroplasty. 3 patients who had needed a cane preoperatively had to use a Zimmer frame or wheelchair for transportation 12 months postoperatively. 4 of the 38 patients who had not required assistance at home preoperatively did so at 12 months, and 3 had had to move to service accommodation.

### Radiographic analysis

13 of the 38 stems were in neutral position in both projections and migrated on average 1.8 mm distally. 5 stems were in varus; 4 of them with the tip directed posteriorly migrated 2.4 mm distally on average. 20 of the stems that were positioned neutrally but with the tip directed posteriorly migrated 2.1 mm distally on average.

4 of the 38 stems were deemed to be too small in the postoperative analyses: 2 in neutral and 2 in varus with the tip posterior. These stems had migrated –3.6, –4.7, –14.8, and –20.7 mm distally, including the 2 outliers. Mean distal migration for the stems that were too small was 11 mm, as compared to 1.9 mm for stems judged to be of the correct size.

8 implanted stems were of smaller size than according to the preoperative plan. These stems subsided 5.7 mm while stems of planned size or larger subsided 2.0 mm.

Median Engh fixation score was 2.5 (2.5–10) at 3, 12, and 24 months, with 1 exception. This patient had a fixation score of –7.5 at 12 months due to a reactive line around the proximal part of the implant. The patient did not report any pain. She died before the 2-year follow-up. Median stability score at 3 months was 9 (3–17); at 12 months it was 3 (2.5–17) and at 24 months it was 3 (3–17).

### RSA

At the last examination, 31 of the 38 stems had migrated distally (> 0.1 mm), the maximum migration being 21 mm. 3 stems had migrated in the anterior direction and 26 in the posterior direction (> 0.2 mm), maximum 4.4 mm. 8 stems had rotated in the anterior direction around the transverse axis and 18 in the posterior direction (> 0.2°), maximum 2°. 27 stems had rotated in retroversion around the longitudinal axis and 1 in anteversion (> 0.7°), maximum 17°. 7 stems had rotated into valgus and 18 into varus (0.2°), maximum 4°.

**Figure 4.**
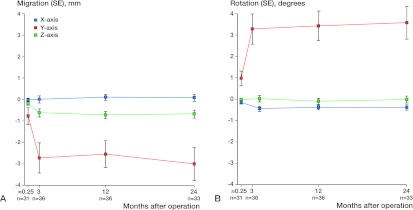
Mean movement. Error bars represent standard error (SE). A. Migration along axis. B. Rotation around axis. Blue: transverse axis; red: longitudinal axis; green: sagittal axis. First examination (0) before weight bearing and 1 week (0.25) before leaving the hospital. Between 3 and 12 months, mean rotation around the sagittal (z-) axis was 0.03–0.11 mm (p < 0.005) towards valgus. Mean migration and rotation in any other direction was not significant (p = 0.09–0.7). Between 12 and 24 months, mean rotation around the longitudinal (y-) axis was 3.4°–3.7° in the dorsal direction (p = 0.02) and mean migration along the transverse (x-) axis was 0.10–0.18 mm in the medial direction (p = 0.02), whereas migration and rotation in any other direction was not significant (p = 0.3–0.9) (t-test for dependent samples).

**Figure 5.**
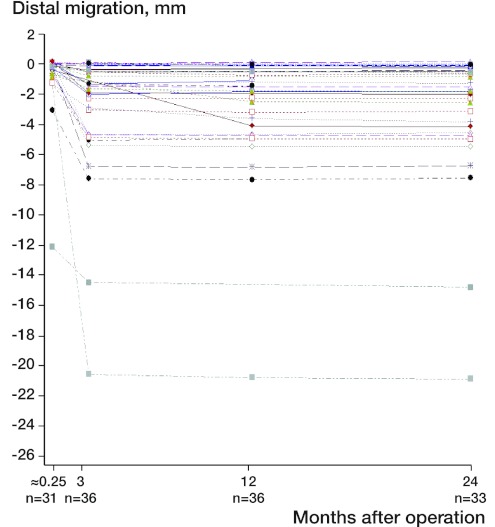
Distal migration: all case profiles.

There were no migrations around the transverse (x-) axis and no rotations around the sagittal (z-) axis, but in all the other directions migration and rotation could be observed up to 3 months (Figure 4). After that point, the stems appeared to stabilize (t-test for dependent samples). Distal migration (y-) was large in some cases, but in these cases it also stopped after 3 months (Figure 5).

### Bone mineral density (BMD) measurements

BMD measurements at the proximal femur were undertaken in 24 women and 11 men who had an unfractured contralateral proximal femur at the time of their index hip fracture. The mean BMD (SD) of the proximal femur was 0.777 (0.10) g/cm^2^ in women and 0.801 (0.13) g/cm^2^ in men. 5 women and 6 men had a proximal femur BMD that was lower than the threshold for osteoporosis (< 0.700 g/cm^2^ in women and < 0.765 g/cm^2^ in men).

**Figure 6.**
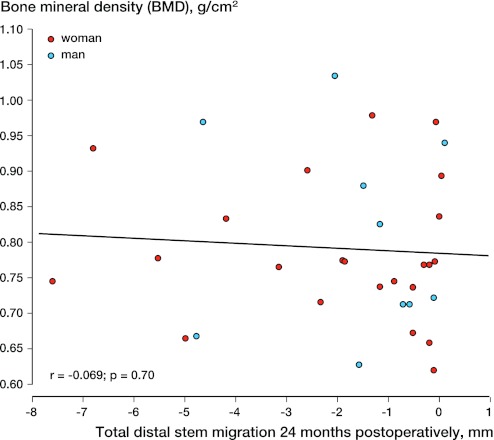
Relationship between bone mineral density of the uninjured proximal femur (measured with DEXA at the time of fracture) and distal stem migration 24 months postoperatively (measured with RSA) in 23 women and 10 men. The linear regression line is solid.

After exclusion of 2 outliers with an implant that was deemed to be too small (with stem migration of 20 and 14 mm, respectively), we found no statistically significant correlation between proximal femur BMD on the contralateral side and distal stem migration at 24 months (r = –0.07, p = 0.70) (Figure 6).

## Discussion

31 of the 38 stems had migrated statistically significantly at 3 months. Stems smaller than according to the preoperative plan and stems deemed too be too small on postoperative radiographs subsided more than those that were of adequate size. There was no correlation between BMD and stem migration, and stem position had no influence on distal stem migration. There were, however, only 5 stems inserted in varus and the trend was that these stems subsided more. The statistically significant differences in rotation and migration occurring after 3 months (Figure 4) were very small, and they were below the limits for statistically significant movement. Thus, we do not consider these differences to be clinically significant.

The rather pronounced initial migration did not result in any clinically adverse effects. At 3 months, our patients reported pain on a VAS to be mean 0.2 (SD 0.5) with no weight bearing and mean 1.5 (SD 2.0) with weight bearing. Both values are lower than the mean of 2.7 (SD 2.6) at 4 months in 906 hemiarthroplasty patients reported in an analysis from the Norwegian Hip Fracture Register (Gjertsen et al. 2008). Patients with femoral implants that subsided for up to 3 months did not (with 1 exception) experience pain, i.e. had less than 40 points for pain in the HHS. This suggests that initial stem migration in this elderly population does not cause any unnatural pain. A large degree of stem migration and rotation cause concern about dislocation, but only 1 hip dislocated early. This was before the patient was mobilized to weight bearing and before RSA examinations could be performed. We have now implanted about 300 Corail prostheses because of dislocated femoral neck fractures, and so far the above-mentioned hip is the only one that has been revised due to dislocation. It is possible that dislocations would have occurred more frequently if a posterior approach had been used, and not the anterior Hardinge approach with the patient supine (Garellik et. al. 2009).

Patients operated with a hemiarthroplasty had a worse Charnley walking score and total HHS than patients with a total hip arthroplasty. However, the pain scores were similar. This difference in functional scores may be explained by the patients operated on with total arthroplasty being younger—even though a recent randomized study by Hedbeck et al. (2011) has indicated that total hip arthroplasty has a better outcome than hemiarthroplasty.

Our results concerning migration are similar to those presented by Sköldenberg et al. (2011) who studied the uncemented fully HA-coated collared Biomet Fracture Stem. However, the stems in our study migrated substantially more in the distal direction and rotated more around the longitudinal axis. In both studies, the stems stabilized after 3 months. These results suggest that a collar applied to the prosthetic stem may be of advantage.

In the RSA study by Campbell et al. (2009) of patients with osteoarthritis who received the same version of the collarless Corail stem, the mean retroversion was half of the values in our elderly fracture population, and distal migration was one third of the corresponding values in the present study. This difference indicates that initial stability with this uncemented collarless stem is more difficult to obtain in an osteoporotic femur, even though our results indicate that the degree of osteoporosis does not matter. After an initial migration, all the extensively HA-coated stems in Campbell's study, Sköldenberg's study, and our study seem to have become firmly embedded, and no adverse effects were observed. Hardy (2010) reported good clinical 10-year results for the collarless Corail stem in 110 patients who were operated on due to a fractured femoral neck.

We did not perform a randomized study comparing the uncemented Corail stem with a cemented prosthesis, because migration patterns would obviously differ. Clinical differences between cemented and uncemented stems in patients with a fractured femoral neck have already been published by Figved et al. (2009). The Swedish (Garellik et al. 2009) and Australian (Graves 2009) hip registries have reported an increased rate of periprosthetic fracture for uncemented stems in this patient group, but no such fractures were observed in our study, although four-fifths of the stems migrated for up to 3 months.
